# The complete mitochondrial genome of *Epeorus herklotsi* (Ephemeroptera: Heptageniidae) and its phylogeny

**DOI:** 10.1080/23802359.2018.1445482

**Published:** 2018-02-28

**Authors:** Xin-Yan Gao, Shu-Sheng Zhang, Le-Ping Zhang, Dan-Na Yu, Jia-Yong Zhang, Hong-Yi Cheng

**Affiliations:** a College of Chemistry and Life Science, Zhejiang Normal University, Jinhua, Zhejiang Province, China;; b Zhejiang Wuyanling National Nature Reserve, Taishun, Zhejiang Province, China;; c Key Lab of Wildlife Biotechnology, Conservation and Utilization of Zhejiang Province, Zhejiang Normal University, Jinhua, Zhejiang Province, China

**Keywords:** Ephemeroptera, mitochondrial genome, *Epeorus herklotsi*, phylogeny

## Abstract

The mitochondrial genome of *Epeorus herklotsi* (Ephemeroptera: Heptageniidae) is a circular molecule of 15,801 bp in length with a base composition of 32.7% A, 32.9% T, 21.5% C, 13.0% G, including extra tRNA^Met^ gene. The *IMQM* tRNA cluster is found in *E. herklotsi* as well as *Parafornuru youi* and two species of *Epeorus* (KM244708, KJ493406), while the typical *IQM* tRNA cluster is found in *Paegniodes cupulatus*. In BI and ML phylogenetic trees, the monophyly of the families Heptageniidae, Baetidae, and Ephemerellidae are highly supported. *E. herklotsi* is a sister clade to *Epeorus* sp2. (KJ493406).

The phylogenetic relationships of Ephemeroptera using morphological or molecular methods still exists disputes (Kristensen 1981; Ogden and Whiting 2003; Zhang et al. 2008; Simon and Hadrys 2013; Li et al. 2014; Misof et al. 2014). Thirteen complete mitochondrial genomes and six partial mitochondrial genomes of Ephemeroptera are available (Zhang et al. 2008; Li et al. 2014; Tang et al. 2014; Zhou et al. 2016). In the 19 known mayfly mitochondrial genomes, gene rearrangements are found in five species (e.g. *Parafronurus youi*, *Epeorus* sp1., *Epeorus* sp2., *Siphluriscus chinensis*, *Alainites yixiani*), especially in Heptageniidae two types of *IQM* and *IMQM* tRNA cluster were found. Hence, we sequenced the mitochondrial genome of *Epeorus herklotsi* (Ephemeroptera: Heptageniidae) to analyse the characteristics of mitochondrial gene arrangement and to discuss the phylogenetic relationships within Ephemeroptera.

The samples of *E. herklotsi* identified by Dr. JY Zhang were collected in Lishui, Zhejiang province, China. All samples of *E. herklotsi* were stored in Lab of HY Cheng, College of Chemistry and Life Science, Zhejiang Normal University. And the total genomic of DNA was isolated from one leg of *E. herklotsi* using Ezup Column Animal Genomic DNA Purification Kit (Sangon Biotech Company, Shanghai, China). The conserved primers and modified primers for PCR amplification were designed according to Zhang et al. (2008). PCR products were sent to Sangon Biotech Company for sequencing with both strands.

The mt genome of *E. herklotsi* is a circular molecule of 15,801 bp in length, with the overall base composition of 32.7% A, 32.9% T, 21.5% C, 13.0% G, including extra *tRNA^Met^* gene. The gene rearrangement with *IMQM* tRNA cluster was found in *E. herklotsi*, which is similar to *P. youi* (Zhang et al. 2008) and two species o*f Epeorus* (KM244708, KJ493406) (Tang et al. 2014), while is different to *Paegniodes cupulatus* with the typical *IQM* tRNA cluster (Zhou et al. 2016).

Bayesian inference (BI) and maximum likelihood (ML) trees were constructed using the 13 PCGs from 20 species using *S. chinensis* (Li et al. 2014) as outgroup (Figure 1). Each alignment was performed by Gblock 0.91b (Castresana 2000) using default settings in order to select conserved regions of the nucleotide. BI and ML analysis were performed by MrBayes3.1.2 (Huelsenbeck and Ronquist 2001) and RAxML 8.2.0 (Stamatakis 2014), respectively. In BI and ML phylogenetic trees, the monophyly of the families Heptageniidae, Baetidae, and Ephemerellidae is highly supported (Figure 1), but the monophyly of Siphlonuridae is failed. *Siphlonurus* sp. (Siphlonuridae) is a clade sister to *Ameletus* sp. (Ameletidae), however, *Siphlonurus immanis* (Siphlonuridae) is a sister clade to *Ephemera orientalis* (Ephemeridae). The monophyly of *Epeorus* is highly supported and *E. herklotsi* is a sister clade to *Epeorus* sp2. (KJ493406). In addition, Baetidae (*Baetis* sp. + *Alainites yixiani*) is a sister clade to Teloganodidae which is not consist with view of Ogden and Whiting (2005). We found the long branch attraction existed in Baetidae, which may affect the phylogenetic relationship of Baetidae.

**Figure 1.**
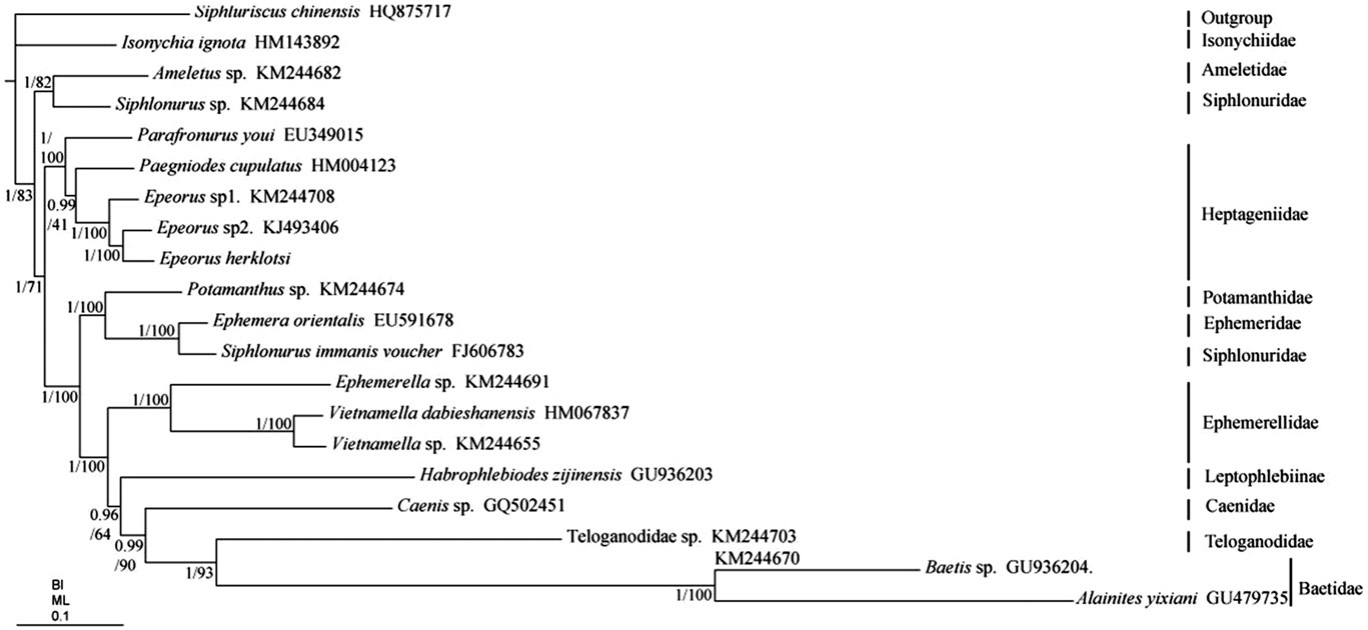
Phylogenetic tree of the relationships among 20 species of Ephemeroptera, including *E. herklotsi* based on the nucleotide dataset of the 13 mitochondrial protein-coding genes. The Bayesian posterior probability values and the maximum-likelihood bootstrap values are indicated above nodes. The GenBank numbers of all species are shown in the figure.
